# The subunit composition of human extracellular superoxide dismutase (EC-SOD) regulate enzymatic activity

**DOI:** 10.1186/1471-2091-8-19

**Published:** 2007-10-15

**Authors:** Steen V Petersen, Zuzana Valnickova, Tim D Oury, James D Crapo, Niels Chr Nielsen, Jan J Enghild

**Affiliations:** 1 Center for Insoluble Protein Structures (inSPIN) and Interdisciplinary Nanoscience Center (iNANO) Department of Molecular Biology, Aarhus University, Gustav Wieds Vej 10C, DK-8000 Aarhus C, Denmark; 2 Department of Pathology, University of Pittsburgh School of Medicine, 3550 Terrace Street, Pittsburgh, PA 15261, USA; 3 Department of Medicine, National Jewish Medical and Research Center, 1400 Jackson Street, Denver, CO 80206, USA; 4 Center for Insoluble Protein Structures (inSPIN) and Interdisciplinary Nanoscience Center (iNANO) at the Department of Chemistry, Aarhus University, Aarhus, Langelandsgade 140, DK-8000 Aarhus C, Denmark

## Abstract

**Background:**

Human extracellular superoxide dismutase (EC-SOD) is a tetrameric metalloenzyme responsible for the removal of superoxide anions from the extracellular space. We have previously shown that the EC-SOD subunit exists in two distinct folding variants based on differences in the disulfide bridge pattern (Petersen SV, Oury TD, Valnickova Z, Thøgersen IB, Højrup P, Crapo JD, Enghild JJ. Proc Natl Acad Sci USA. 2003;100(24):13875–80). One variant is enzymatically active (aEC-SOD) while the other is inactive (iEC-SOD). The EC-SOD subunits are associated into covalently linked dimers through an inter-subunit disulfide bridge creating the theoretical possibility of 3 dimers (*aa*, *ai *or *ii*) with different antioxidant potentials. We have analyzed the quaternary structure of the endogenous EC-SOD disulfide-linked dimer to investigate if these dimers in fact exist.

**Results:**

The analyses of EC-SOD purified from human tissue show that all three dimer combinations exist including two homo-dimers (*aa *and *ii*) and a hetero-dimer (*ai*). Because EC-SOD is a tetramer the dimers may combine to generate 5 different mature EC-SOD molecules where the specific activity of each molecule is determined by the ratio of aEC-SOD and iEC-SOD subunits.

**Conclusion:**

This finding shows that the aEC-SOD and iEC-SOD subunits combine in all 3 possible ways supporting the presence of tetrameric enzymes with variable enzymatic activity. This variation in enzymatic potency may regulate the antioxidant level in the extracellular space and represent a novel way of modulating enzymatic activity.

## Background

Superoxide dismutase enzymes (SOD; EC 1.15.1.1) are a family of metalloenzymes that converts the superoxide radical to hydrogen peroxide and water. Two copper/zinc-containing isoforms of SOD exists in mammals including Cu/Zn-SOD (SOD1) located in intracellular compartments [1-3] and extracellular SOD (EC-SOD; SOD3) found predominantly in the extracellular matrix of tissues [4-7]. Cu/Zn-SOD is a 32 kDa homo-dimer [8,9], whereas EC-SOD is a 135 kDa tetrameric glycoprotein with regional amino acid sequence homology to Cu/Zn-SOD [10,11]. The N-terminal region of EC-SOD is involved in hydrophobic inter-subunit interactions stabilizing the EC-SOD tetramer [12,13]. The importance of these interactions has been emphasized by the finding that the dimeric rat EC-SOD [14], is converted to a tetramer by substituting a hydrophilic residue (Asp) within the N-terminal region for a hydrophobic one (Val) [15]. In addition, the reverse substitution converted tetrameric mouse EC-SOD into a dimer. The C-terminal region of EC-SOD contains a cluster of basic amino acid residues [11] with affinity for heparin/heparan sulfate [16,17] and type I collagen [18,19]. We have previously shown, that this region can be proteolytically removed before secretion in a two-step process involving both an endoproteinase and a carboxypeptidase [20-22]. This cleavage event changes the affinity for extracellular matrix (ECM) components and affects the biodistribution of the protein. Moreover, the cystein residue responsible for an inter-subunit disulfide bridge is present in this region. When mature EC-SOD is analyzed under denaturing conditions, cleaved subunits will therefore appear as monomers while intact subunits will be dimers [23].

The overall structure of EC-SOD has not been determined. However, we have shown that human EC-SOD exists as two different folding variants with distinct disulfide bridge patterns [23,24]. One variant has SOD activity (aEC-SOD) whereas the other form (iEC-SOD) lacks the capacity to dismutate the superoxide radical. While the disulfide bridge pattern of aEC-SOD encompass a conserved disulfide bridge found in all Cu/Zn-containing SODs [25] this disulfide bridge is absent in iEC-SOD. This highly conserved disulfide bridge is essential for maintaining the catalytic activity supporting the absence of enzymatic activity in iEC-SOD. Interestingly, EC-SOD from some species including rabbit, mouse, and rat lack a cysteine residue essential for the formation of iEC-SOD and we have shown that rabbit EC-SOD indeed exists only as aEC-SOD [24]. As a consequence of this, the specific activity of rabbit EC- SOD was found to be 2-fold higher relative to the human protein [24].

Disulfide bridges have generally been categorized as important for structural stability or catalytic activity in, e.g., thiol-disulfide oxidoreductases as thioredoxin and protein disulfide isomerase, where cysteine residues are directly involved in catalysis. However, a third function of disulfide bridges is becoming evident, which involves allosteric regulation of protein activity by reduction and oxidation of disulfide bonds with no catalytic activity [26]. It is intriguing to speculate that the presence of two disulfide variants in human EC-SOD may represent an example of an allosteric switch.

We have previously discussed the possibility that human EC-SOD molecules exist where the ratio of aEC-SOD and iEC-SOD subunits within the tetramer varies to produce molecules with different specific activities [23]. Here we show, that this is indeed the case as the EC-SOD dimer can be separated into three distinct pools representing the *aa *and *ii *homo-dimers and the *ai *hetero-dimer. The existence of disulfide dependent folding variants of the same protein has not been observed before and because of the impact on the enzymatic activity it may represent a novel way of regulating the enzymatic activity of EC-SOD *in vivo*.

## Results

### Electrophoretic mobility of the EC-SOD dimer

**Figure 1:**
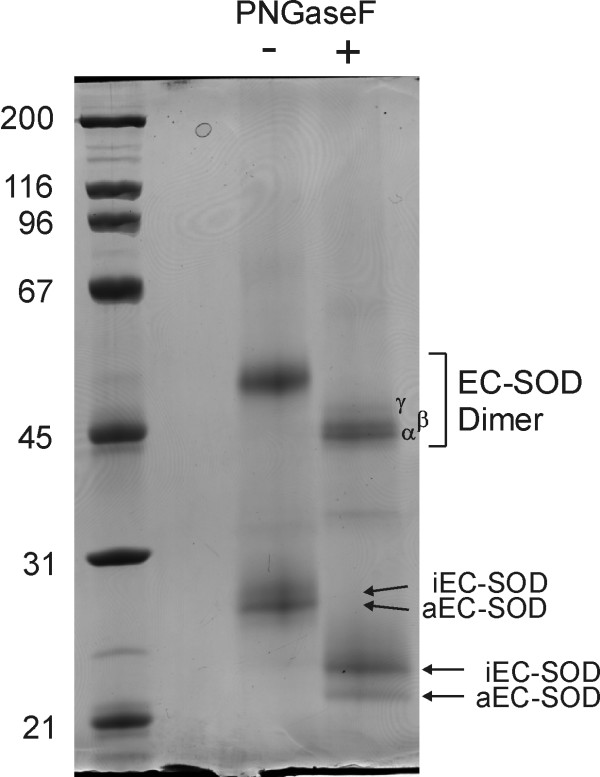
**The EC-SOD dimers separate into three forms**. Human EC-SOD was subjected to non-reduced SDS-PAGE using a 9% acrylamide gel. Glycosylated EC-SOD (-PNGase F) separates into the disulfide-linked dimer and monomers. The monomers are resolved into aEC-SOD and iEC-SOD as previously shown [23]. The dimer migrates as a fuzzy band of ~52 kDa. When EC-SOD is deglycosylated (+PNGase F) the size of the monomers and dimer is reduced consistent with the removal of a single glycan in each subunit. The deglycosylated dimer resolved into three closely migrating bands of 45 – 49 kDa denoted α, β, and γ as indicated. The position of the deglycosylated monomers is indicated. The gel was stained by Coomassie brilliant blue. A molecular weight marker is indicated on the left. Deglycosylation of EC-SOD improved the separation in non-reduced SDS-PAGE and revealed that the disulfide-bonded dimer migrates as 3 bands most likely caused by differences in folding.

The C-terminally cleaved EC-SOD subunit is lacking the inter-chain disulfide bond and is easily separated from the intact disulfide-bonded EC-SOD dimer during non-reducing SDS-PAGE. We have previously analyzed the C-terminally cleaved EC-SOD subunit and shown the two folding variants are resolved when using optimal electrophoretic conditions [23] (Figure 1). However, the disulfide-bonded EC-SOD dimer remained poorly resolved and migrated as a broad band (Figure 1). Although the *N*-linked glycan at Asn89 is homogenous [16,27] deglycosylation significantly improved the resolution in unreduced SDS-PAGE resulting in two closely spaced bands that we denoted α and β, and a slightly slower migrating band called γ (Figure 1). Similarly, the folding variants of the deglycosylated C-terminally cleaved EC-SOD subunit was better resolved by SDS-PAGE. This result shows that the EC-SOD dimer exists in three distinct populations which can be separated by SDS-PAGE following deglycosylation.

### Separation of EC-SOD dimers by reverse-phase HPLC

**Figure 2:**
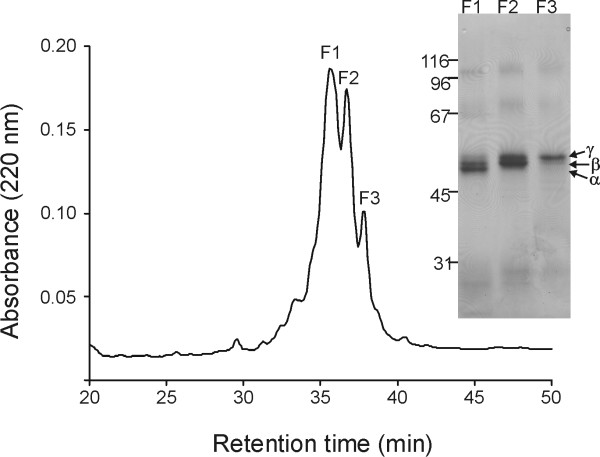
**The EC-SOD dimers can be separated by reverse-phase HPLC**. EC-SOD can be separated into monomer and dimer by reverse-phase HPLC [23]. The isolated dimer was subsequently denatured and alkylated using iodoacetamide. The alkylated material was subjected to reverse-phase HPLC using a C_8 _column. The material separated into three major peaks without base-line separation. The collected fractions were denoted F1, F2, and F3 as indicated. The material was subsequently analyzed by non-reducing SDS-PAGE using a 9% acrylamide gel (inset). Protein was visualized by silver staining. Three distinct bands were detected and denoted α, β, and γ according to figure 1. A molecular weight marker is indicated on the left.

The C-terminally cleaved EC-SOD subunit and disulfide-linked dimer can be separated by reverse-phase HPLC [23]. Moreover, the C-terminally cleaved EC-SOD subunit can be further separated into the two different folding variants by reverse-phase HPLC following denaturation and *S*-carboxyamidomethylation [23]. To investigate whether the three electrophoretic variants of dimeric EC-SOD observed by SDS-PAGE could similarly be resolved, we subjected alkylated dimeric EC-SOD to reverse-phase analysis using an octyl-derivatized solid support. The material was found to separate into three major peaks without baseline separation (Figure 2) and fractions were collected manually to reduce cross contamination and following subjected to SDS-PAGE analysis (Figure 2, inset). This analysis revealed that fraction 1 contained components α and β; fraction 2 contained components β and γ; and fraction 3 was found to contain component γ. Based on this pattern of separation, it is likely that baseline separation would have produced three distinct fractions containing isolated components. We conclude that the 3 disulfide-bonded EC-SOD variants designated α, β and γ can be separated by reverse-phase HPLC and that these, based on the difference in electrophoretic mobility, represent folding variants.

### Analysis of dimeric components by tryptic cleavage

**Figure 3:**
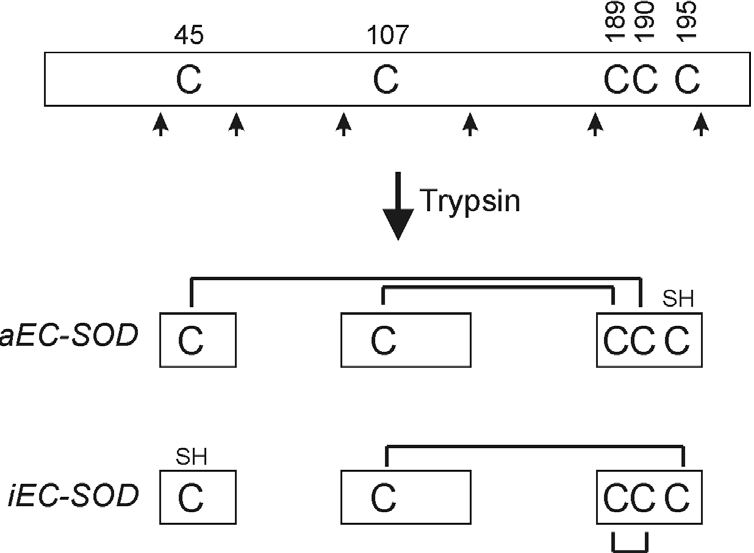
**Schematic representation of disulfide-linked tryptic peptides**. A representation of human EC-SOD is depicted with the cysteine residues involved in intramolecular disulfide bonding indicated and tryptic cleavage sites generating cysteine-containing peptides shown by arrows. Following cleavage by trypsin, aEC-SOD is represented by a disulfide-linked triple peptide and iEC-SOD represented by a disulfide-linked double peptide. These two structures can be separated by RP-HPLC and can thus be used to detect the presence of the two folding variants.

**Figure 4:**
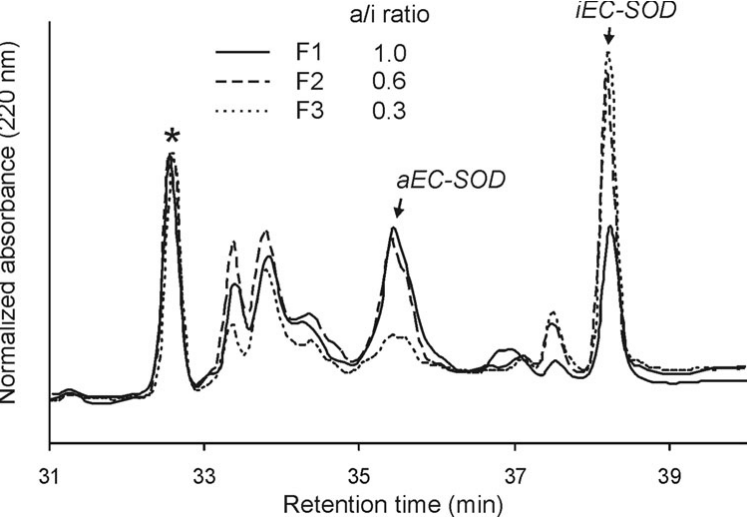
**Tryptic fingerprints indicate both EC-SOD homo- and hetero-dimers**. The partially separated EC-SOD dimers were subjected to trypsin cleavage and the generated peptides isolated by reverse-phase HPLC. Two peaks indicate the presence of aEC-SOD (35.5 min) or iEC-SOD (38.2 min) as indicated. Three data sets were collected: F1, solid line; F2, dashed line; F3, dotted line) and normalized using the peak representing Val24-Arg34 (*). The yield of this peptide did not differ between aEC-SOD and iEC-SOD. The level of the two folding variants could be estimated by measuring the ratio of absorbance between the peaks representing aEC-SOD and iEC-SOD and the Val24-Arg34 peptide. The obtained ratio is indicated.

The folding variants of EC-SOD can be identified by characterizing the peptides from unreduced tryptic digests (Figure 3) [23]. The isolated EC-SOD dimers were analyzed in the same way (Figure 4) and relevant peaks were identified by MALDI-MS (data not shown). The peptide Val24-Arg34 (denoted by *, Figure 4) is unaffected by the Cys connectivity and was used to normalize the chromatograms to determine the ratio of aEC-SOD and iEC-SOD. This ratio does not reflect the molar level between the two folding variants, but is used as a simple measure to compare the amounts of the two variants in the collected fractions. The peptide fingerprint of the material collected in fraction 1, 2, and 3 present aEC-SOD/iEC-SOD-ratios of 1.0, 0.6, and 0.3, respectively (Figure 4). Taking the lack of base-line separation into consideration, the analyses thus shows that the dimer collected in fraction 1 is likely to represent a homo-dimer composed of aEC-SOD (see Figure 2, band α). Fraction 2 represents a hetero-dimer composed of aEC-SOD and iEC-SOD (Figure 2, band β), whereas fraction 3 represents a homo-dimer composed of iEC-SOD subunits only (Figure 2, band γ).

## Discussion

We have previously analyzed purified human EC-SOD subunits and shown that they fold into two distinct molecules based on different disulfide-bridge connectivity [23]. To simplify the analysis and data interpretation, the initial study focused on determining the disulfide connectivity of the subunits isolated by reverse phase HPLC. We determined that the EC-SOD subunit folds into two distinct variants called aEC-SOD (enzymatically *active*) and iEC-SOD (enzymatically *inactive*) [23]. However, from this study it could not be determined if the folding differences were compatible with the formation of homo- and/or hetero-dimers. To investigate this, we have in the present study purified and analyzed the disulfide-linked EC-SOD dimer. During the purification and analyses, rigorous measures were taken to eliminate disulfide bridge reshuffling, since both aEC-SOD and iEC-SOD contains one free cysteine residue [23]. The purification of EC-SOD from human aorta was conducted in the cold room using neutral pH buffers and conditions compatible with maintaining the native structure. Subsequent fractionation by RP-HPLC was performed at low pH where disulfide bridge exchange is inhibited since the pKa value of a typical thiol group is in the range of 8.3 [28]. In addition, prior to the separation of the disulfide-bonded dimers by RP-HPLC, the protein is denatured in the presence of iodoacetic acid. Moreover, we have previously shown that both iEC-SOD and aEC-SOD subunits are present in whole tissue homogenates prepared in the presence of iodoacetamide [23]. The identification of the three different EC-SOD dimers reported in this paper is therefore not the result of disulfide bridge exchange reactions.

### Dimers can support the generation of five tetramers with variable activity

**Figure 5:**
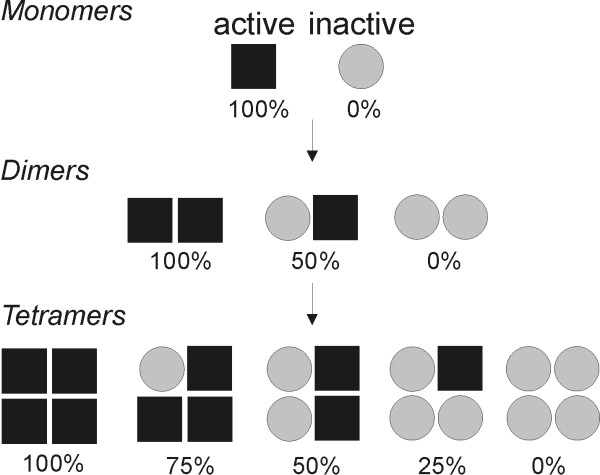
**Schematic representing the antioxidant potential of EC-SOD**. The EC-SOD subunit exists as an active folding variant with SOD activity (aEC-SOD; filled squares) and an inactive (iEC-SOD; gray circles). Our results show that the two different subunits can combine to form dimers with variable activities. Tetrameric EC-SOD is maintained by interactions within the N-terminal region, which are likely to be unaffected by the folding of the catalytic domain. We thus propose that dimers combine to generate five different tetramers with distinct SOD activities. The relative SOD activity of each structural level is indicated below each structure. The presence or absence of the ECM-binding regions is not indicated, as this is likely not affecting the tetramer assembly.

Structural considerations suggest that the EC-SOD tetramer is likely to be organized as two interacting dimers [15]. The characterization of the isolated EC-SOD dimer is thus likely to be relevant for the quaternary structure of the intact EC-SOD molecule. We show that the population of authentic EC-SOD molecules purified from human aorta is composed of three different dimers providing evidence for the formation of EC-SOD molecules with variable degrees of enzymatic activity (Figure 5). The purification of the EC-SOD tetramer subclasses is likely to be difficult due to the similar biochemical properties of such molecules. However, it is the N-terminal regions that are responsible for the formation of the EC-SOD tetramers [12] and they are likely not affected by the difference in folding of the catalytic region. It is thus plausible that the identified dimers are able to assemble into tetramers in a random fashion and generate five different EC-SOD tetramers with variable SOD activity as previously hypothesized (see Figure 5) [23].

### Spatial interactions between homo- and hetero-dimers are likely to be different

Structural studies have shown that the Cu/Zn-SOD homo-dimer is stabilized by inter-subunit hydrogen bonds between the highly conserved residues Gly51, Gly114, and Ile151 (amino acid numbering refers to human Cu/Zn-SOD) [8,25]. Similarly, these residues are involved in forming hydrogen bonds in the transient hetero-dimer composed of the Cu/Zn-SOD copper chaperone (CCS) and Cu/Zn-SOD during biosynthesis [29]. In addition to the inter-subunit hydrogen bonds, the Cys57–Cys147 disulfide bridge function as a mediator of contacts points in the Cu/Zn-SOD homo-dimer [25]. The amino acid sequence of human EC-SOD is consistent with the presence of similar inter-subunit hydrogen bonds within the EC-SOD disulfide-bonded dimer [11], however, the homologous disulfide bridge is not present in iEC-SOD [23]. Based on this, the contacts within the iEC-SOD homo-dimer and the *ai *hetero-dimer are likely to be different from the interactions described for the Cu/Zn-SOD homodimer. This implies that the EC-SOD subunits function independently of inter subunit interactions described for Cu/Zn-SOD.

## Conclusion

Our data show that the two folding variants of EC-SOD are able to combine and form dimers and most likely also tetramers exhibiting variable SOD activity. The level of SOD activity in the extracellular space can thus be regulated by varying the ratio of aEC-SOD and iEC-SOD subunits in the tetramer. The mechanism responsible for this unique control of the enzymatic activity is not understood. However, it is interesting to note that the level of Cu/Zn-SOD activity can be regulated post-translationally by the activation of a preexisting apo-pool of Cu/Zn-SOD [30]. This activation mechanism is mediated by the activation of the copper chaperone for Cu/Zn-SOD (CCS). We speculate that the existence of active and inactive EC-SOD folding variants may similarly be regulated in response to oxidative stress either during folding in the endoplasmatic reticulum or via a post-translational allosteric regulation of disulfide connectivity. The present data suggests that SOD activity of the EC-SOD tetramer is dynamic. It will be of great importance to determine how EC-SOD activity is regulated and define the role this plays in modulating the function and toxicity of oxidant radicals in extracellular spaces.

## Methods

### Proteins

EC-SOD was purified from human aorta as previously described, except that the cation exchange chromatography step was omitted [31]. PNGase F was from Roche and sequence grade porcine trypsin was purchased from Promega.

### Polyacrylamide gel electrophoresis

Proteins were separated by sodium dodecyl sulfate-polyacrylamide gel electrophoresis (SDS-PAGE) using 9% uniform gels and the glycine/2-amino-2-methyl-1,3-propanediol-HCl system as described [32]. Prior to electrophoresis, samples were boiled in the presence of 1% SDS. For reducing conditions, 30 mM dithiotreitol (DTT) was included.

### Deglycosylation of EC-SOD

Approximately 5 mg of EC-SOD in 50 μl of 50 mM Na_2_HPO_4_, 0.1% (w/v) SDS (pH 7.4) was boiled for 5 min and allowed to cool before the addition of 0.75% (v/v) Triton ×-100 and 2 units of PNGase F. The sample was incubated at 37°C for 3 h before the reaction products were analyzed by SDS-PAGE.

### Separation of EC-SOD folding variants

The monomers and disulfide-linked dimers of EC-SOD were separated by reverse-phase HPLC as previously described [23]. Briefly, ~60 μg EC-SOD in Tris-HCl and NaCl was acidified by the addition of trifluoroacetic acid (TFA) and applied to a 2.1 mm × 220 mm Aquapore RP-300 C_8 _reverse-phase HPLC column (Brownlee Labs) connected to an ÄKTAexplorer system (GE Healthcare). Bound proteins were eluted by a gradient of solvent B (90% acetonitrile, 0.08% TFA) in solvent A (0.1% TFA) (6% B min^-1^). The column was operated at 23°C at a flow rate of 200 μl min^-1^. Protein was detected at 220 and 280 nm and fractions collected manually. The fraction containing dimeric EC-SOD was subsequently lyophilized and redissolved in 30 mM HEPES (pH 8.3) containing 5 M guanidinium hydrochloride and 25 mM iodoacetamide. The reaction was performed at 23°C for 30 min. The material was subsequently acidified by addition of TFA and the alkylated dimers separated by reverse-phase HPLC as described above.

### Tryptic digestion of separated EC-SOD dimers

The collected fractions containing *S*-carboxyamidomethylated dimeric EC-SOD was digested overnight at 37°C in 0.5 M HEPES (pH 8.3) using porcine trypsin with an approximate weight ratio of 1:20. The sample was subsequently lyophilized and rehydrated in 0.1% TFA. The tryptic peptides were separated by reverse-phase HPLC using a linear gradient of 1% B min^-1 ^using the HPLC system described above. Fractions were collected manually.

### Mass spectrometric analysis

Peptides were analyzed by MALDI-MS using a quadropole/time-of-flight (Q-TOF) *Ultima *Global mass spectrometer (Micromass) and α-cyano-4-hydroxycinnamic acid (Sigma) as the matrix. Prior to analysis, the mass spectrometer was calibrated using a mixture of PEG-200, -600, -1000, -2000 and NaI. Identification of the peptides was performed by using the GPMAW software [33].

## List of abbreviations

EC-SOD, extracellular superoxide dismutase; ECM, extracellular matrix; PNGase F, peptide *N*-glycosidase F.

## Competing interests

The author(s) declares that there are no competing interests.

## Authors' contributions

SVP performed the experiments and drafted the manuscript; ZV purified human EC-SOD; TDO, JDC and NCN participated in the design of the study; JJE conceived the study and participated in the design. All authors read and approved the final manuscript.
